# Supplementary figures and images for: Pathogenic strains of Shewanella putrefaciens contain plasmids that are absent in the probiotic strain Pdp11
Source: PeerJ. 2022 Oct 24;10:e14248. doi: 10.7717/peerj.14248 (PMC9610664; doi:10.7717/peerj.14248)

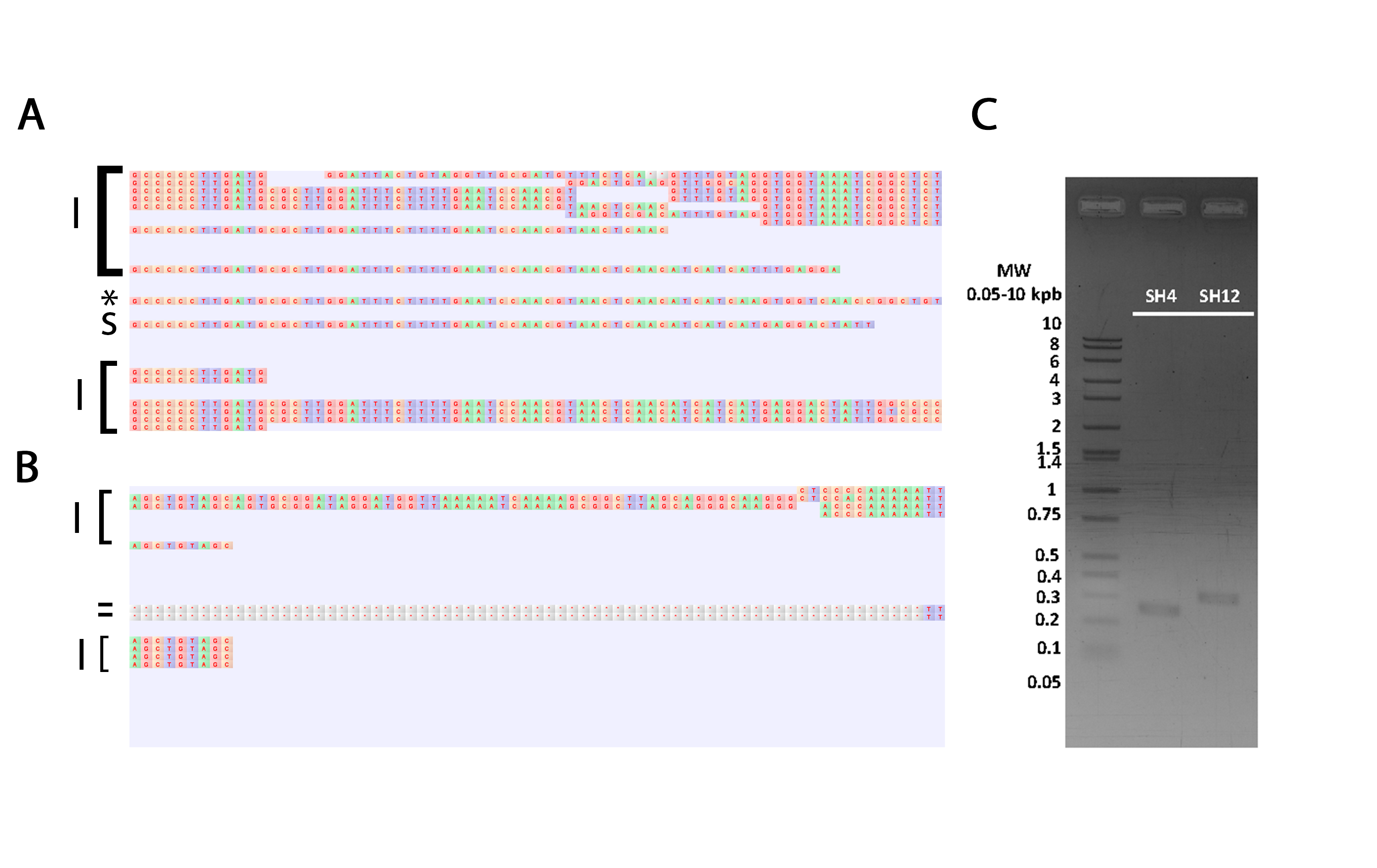

Supplement: Supplemental Information 1 — (A) Representation of the complete pSH4 non-validated region mapping by Illumina (I), Sanger (S) and PCR sequences over pSH4 consensus region and the asterisk indicated the PCR results that confirm the non-validated region. In pSH4 the gap region was confirm by one PCR sequence and more than 20 Illumina sequence (B) Representation of the complete pSH12 non-validated region mapping by Illumina, Sanger and PCR sequence over pSH12 consensus region and the hyphen indicated the Sanger gaps and the no confirm region. The pSH4 gap was support only for first two short illumine ending, this no confirm the presence of this region in the SH12 plasmid (C) Agarose gel electrophoresis of amplicons from both, pSH4 and pSH12 plasmids non-validated regions. [file peerj-10-14248-s001.png]

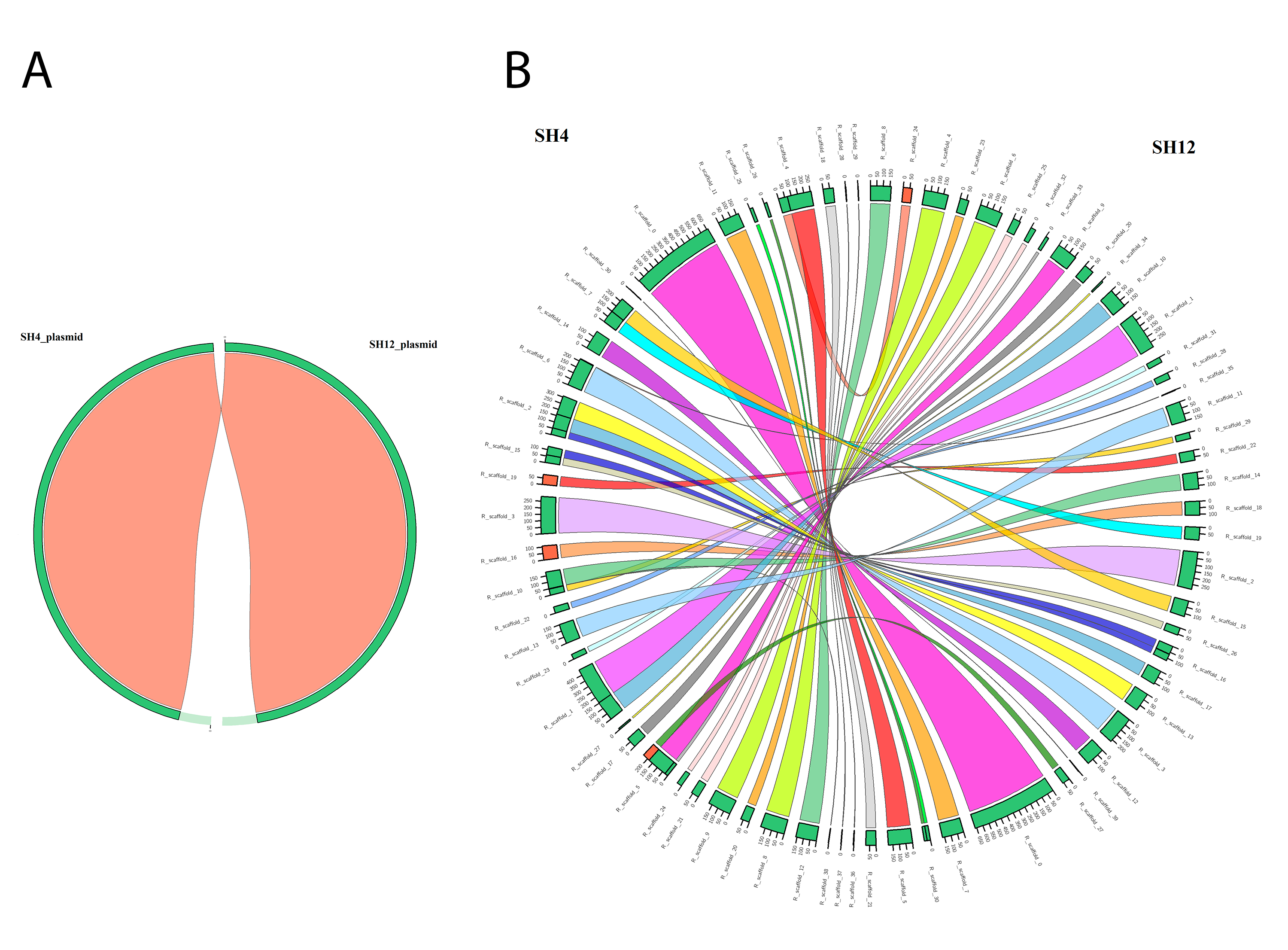

Supplement: Supplemental Information 2 — Both diagrams shows the pairwise multiple synteny blocks between SH4 and SH12 strains at plasmid (A) and genomic (B) level, with no synteny differences in case of plasmid because of the high similarity (94.24%), and multiple syntenic regions with high similarities (99.42%) distributed in 58 and 46 scaffolds in SH12 and SH4 genomes, respectively. The syntenic diagrams were generated using Circos as an inbuilt tool of Sibelia. [file peerj-10-14248-s002.png]
