# Supplementary material for: Pathogenic strains of Shewanella putrefaciens contain plasmids that are absent in the probiotic strain Pdp11
Source: PeerJ. 2022 Oct 24;10:e14248. doi: 10.7717/peerj.14248 (PMC9610664; doi:10.7717/peerj.14248)
Supplement: Supplemental Information 3 — a + and − indicate the presence or absence of plasmids, respectively. b optimum conditions [file peerj-10-14248-s003.docx]

| **Presence of plasmid**^a^ | | | | | | | | | | |
| --- | --- | --- | --- | --- | --- | --- | --- | --- | --- | --- |
| **Strains** | **Culture medium/Temperature/time** | | | | | | | | | |
|  | **TSBs/23/24^b^** | **TSBs/23/48** | **TSBs + glycerol/23/24** | **TSBs + glycerol/23/48** | **TSBs/4/24** | **TSBs/4/48** | **TSBs + glycerol/4/24** | **TSBs + glycerol/4/48** | **M9/23/24** | **M9/23/48** |
| **SH4** | + | + | + | + | + | + | + | + | + | + |
| **SH6** | - | - | - | - | - | - | - | - | - | - |
| **SH12** | + | + | + | + | + | + | + | + | + | + |
| **SH16** | - | - | - | - | - | - | - | - | - | - |
| **SH19** | - | - | - | - | - | - | - | - | - | - |
| **Pdp11** | - | - | - | - | - | - | - | - | - | - |
| **SdM1** | - | - | - | - | - | - | - | - | - | - |
| **SdM2** | - | - | - | - | - | - | - | - | - | - |
