# Supplementary material for: Pathogenic strains of Shewanella putrefaciens contain plasmids that are absent in the probiotic strain Pdp11
Source: PeerJ. 2022 Oct 24;10:e14248. doi: 10.7717/peerj.14248 (PMC9610664; doi:10.7717/peerj.14248)
Supplement: Supplemental Information 4 — a ORFs were named as ORFn followed by the plasmid name (pSH12 or pSH4), where n is the ORF id. [file peerj-10-14248-s004.docx]

| Strain | ORF^a^ | Strand | Start | Stop | Lenght (nt\|aa) | G+C content (%) |
| --- | --- | --- | --- | --- | --- | --- |
|  | 1 | + | 1524 | 1874 | 351 \| 116 | 46.44 |
|  | 2 | + | 43 | 306 | 264 \| 87 | 36.74 |
|  | 3 | + | 2109 | 2420 | 312 \| 103 | 41.67 |
| SH4 | 4 | - | 2006 | 1827 | 180 \| 59 | 44.35 |
|  | 5 | - | 1541 | 1158 | 384 \| 127 | 44.15 |
|  | 6 | - | 1288 | 728 | 561 \| 186 | 44.78 |
|  | **7** | - | 727 | 275 | 453 \| 150 | 57.22 |
|  | 1 | + | 1588 | 1908 | 351 \| 116 | 36.74 |
|  | 2 | + | 2143 | 2454 | 312 \| 103 | 42.47 |
| SH12 | 3 | + | 204 | 467 | 264 \| 87 | 41.67 |
|  | 4 | - | 2040 | 1861 | 180 \| 59 | 48.76 |
|  | 5 | - | 1575 | 889 | 687 \| 228 | 46.44 |
|  | 6 | - | 888 | 436 | 453 \| 150 | 48.76 |
